# ANP32B Is a Nuclear Target of Henipavirus M Proteins

**DOI:** 10.1371/journal.pone.0097233

**Published:** 2014-05-13

**Authors:** Anja Bauer, Sebastian Neumann, Axel Karger, Ann-Kristin Henning, Andrea Maisner, Boris Lamp, Erik Dietzel, Linda Kwasnitschka, Anne Balkema-Buschmann, Günther M. Keil, Stefan Finke

**Affiliations:** 1 Friedrich-Loeffler-Institut, Federal Research Institute for Animal Health, Institute of Molecular Biology, Greifswald – Insel Riems, Germany; 2 Friedrich-Loeffler-Institut, Federal Research Institute for Animal Health, Institute of Novel and Emerging Infectious Diseases, Greifswald – Insel Riems, Germany; 3 Institute of Virology, Philipps-University Marburg, Marburg, Germany; International Centre for Genetic Engineering and Biotechnology, Italy

## Abstract

Membrane envelopment and budding of negative strand RNA viruses (NSVs) is mainly driven by viral matrix proteins (M). In addition, several M proteins are also known to be involved in host cell manipulation. Knowledge about the cellular targets and detailed molecular mechanisms, however, is poor for many M proteins. For instance, Nipah Virus (NiV) M protein trafficking through the nucleus is essential for virus release, but nuclear targets of NiV M remain unknown. To identify cellular interactors of henipavirus M proteins, tagged Hendra Virus (HeV) M proteins were expressed and M-containing protein complexes were isolated and analysed. Presence of acidic leucine-rich nuclear phosphoprotein 32 family member B (ANP32B) in the complex suggested that this protein represents a direct or indirect interactor of the viral matrix protein. Over-expression of ANP32B led to specific nuclear accumulation of HeV M, providing a functional link between ANP32B and M protein. ANP32B-dependent nuclear accumulation was observed after plasmid-driven expression of HeV and NiV matrix proteins and also in NiV infected cells. The latter indicated that an interaction of henipavirus M protein with ANP32B also occurs in the context of virus replication. From these data we conclude that ANP32B is a nuclear target of henipavirus M that may contribute to virus replication. Potential effects of ANP32B on HeV nuclear shuttling and host cell manipulation by HeV M affecting ANP32B functions in host cell survival and gene expression regulation are discussed.

## Introduction

Hendra and Nipah viruses (HeV and NiV, genus henipavirus) are highly pathogenic zoonotic paramyxoviruses [1], [2]. Though fruit bats are natural hosts for henipaviruses, spill-over to pigs and horses and subsequent transmission to humans cause severe respiratory and neurological diseases with high fatality rates [3]. All six henipavirus proteins are essentially involved in cytoplasmic virus replication and virion release at the plasma membrane [4].

Several henipavirus proteins have been extensively studied for their ability to interact with cellular partners to support virus infection. As an attachment protein, the glycoprotein G binds to conserved cellular ephrin B2 or B3 receptors and supports the ability of henipaviruses to infect a broad spectrum of host cells from different species [5]. This step is followed by the fusion of the cellular and virus membrane, which is mediated by the fusion protein F [6]. As many other paramyxovirus phosphoproteins, P together with accessory V, W and C proteins represent type 1 interferon antagonists and inhibit both beta-interferon induction and downstream interferon responses (reviewed in [7]). Though interferon regulation and receptor usage contribute to henipavirus replication and pathogenesis, it still is an open question whether other virus-host interactions exist that either target cellular proteins as agonists of virus replication or interfere with antiviral responses.

Another virus protein that could be involved in molecular virus - host interactions is the structural matrix protein M. According to the well-known function of M proteins in paramyxovirus assembly, henipavirus M proteins are essentially involved in membrane envelopment and budding [8]. In NiV M, a late domain like YMYL and a YPLGVG motif contribute to budding activity. Interestingly, deletion of the YMYL or YPLGVG motifs led to a re-distribution of NiV M to the nucleus [9], [10]. Although henipaviruses replicate in the cytoplasm, the structural M protein comprises positively charged nuclear localization signals (NLS) and leucin-rich nuclear export sequences (NES). Accordingly, nucleo-cytoplasmic trafficking of NiV M has been observed [11]. Furthermore, Wang and colleagues identified a highly conserved lysine residue within the NLS of NiV M that is involved in nuclear import and also in nuclear export regulation by serving as a potential mono-ubiquitinylation site. Moreover, the release of virus like particles (VLPs) depends on nucleo-cytoplasmic trafficking. This indicates that virus-cell interactions in the nucleus are involved in efficient virus morphogenesis and release [11]. Molecular targets of nuclear M and a detailed mechanism behind it, however, are unknown.

M proteins of other cytoplasmatically replicating negative strand RNA viruses also enter the nucleus [12]–[16]. Accumulation of Vesicular Stomatitis Virus (VSV; *Rhabdoviridae*) M in the nucleus not only inhibits nuclear export of cellular mRNAs [17], but also inhibits cellular transcription [18], [19], both contributing to an efficient host cell shut off in VSV infections. Respiratory Syncytial Virus (RSV; *Paramyxoviridae*) also affects host cell transcription, suggesting that RSV M is involved in host cell shut off similar to VSV [15], [20]. Nuclear functions of M proteins from other paramyxoviruses or rabies virus (*Rhabdoviridae*) remain unknown [11], [16], [20]. Likewise, it is also not clear, whether nuclear-cytoplasmic trafficking of NiV M is only needed to acquire post-translational modifications or whether also nuclear structures are targeted to interfere with cellular functions. Similar to RSV [21], chromosome region maintenance 1 (Crm1)-dependent export has been proposed for NiV M [11]. Crm1 mediates nuclear export of proteins and RNA molecules in a Ran-GTP dependent manner (reviewed in [22]).

Whereas most proteins directly bind to the Crm1, Crm1-mediated export of RNAs relies on variable adaptors, for example ANP32B (Acidic leucine-rich nuclear phosphoprotein 32 family member B). ANP32B, also designated as PHAPI2 or SSP29, binds to Hu-antigen R (HuR)-mRNA complexes and thus recruits specific mRNAs to the Crm1 export machinery. Interestingly, stimulus driven export of CD83 mRNAs in dendritic cells [23] and export of foamy virus are ANP32B-dependent [24]. Beside retroviral RNAs, also virus proteins have been found to interact with ANP32B, as demonstrated for the Rep68 protein of adeno-associated virus [25]. In addition to the adaptor function of ANP32B in Crm1-dependent nuclear export, ANP32B is also known to regulate cellular promoters [26] and, as a direct caspase-3 substrate, inhibits caspase-3 dependent apoptosis induction [27]. Interestingly, interaction of ANP32B with.

In order to assess whether cellular proteins of the nuclear export machinery or any other nuclear structures are targeted by henipavirus M proteins, protein complexes containing affinity-tagged HeV matrix protein were isolated and analyzed for cellular M-binding proteins by mass spectrometry. ANP32B was identified as a possible candidate and the impact of ANP32B on the nuclear-cytoplasmic trafficking of HeV and NiV M proteins was analysed in transfected and in virus infected cells.

## Materials and Methods

### Cells

HEK293T cells (Collection of Cell Lines in Veterinary Medicine, Friedrich-Loeffler-Institut, Insel Riems, Germany) were cultivated in Minimal Essential Medium (Earl’s and Hank’s salts 1∶1) supplemented with non-essential amino acids and 10% fetal calf serum. The human alveolar epithelial cell line A549 (ATCC; CCL-185) was cultivated in Dulbecco’s Modified Eagle Medium supplemented with 10% fetal calf serum. HEK293T cell lines stably expressing shRNAs directed against human ANP32B mRNA (shRNA 765, shRNA 767) or irrelevant murine ASS1 (argininosuccinate synthase 1) mRNA (shRNA 782 and shRNA 784) were generated by transfection of corresponding shRNA-encoding pGIPZ vectors (Thermo Scientific; clone IDs V3LHS_353559, V3LHS_353558, V3LMM_518443 and V3LMM_518446, respectively) and selection of puromycin resistant cells.

### Expression Plasmids

Expression plasmids for HeV M were generated by RT-PCR amplification of the HeV M coding sequence from purified Hendra Virus RNA (H. Weingartl, National Centre for Foreign Animal Disease, Canada) with primers HeV MXma/HeV MNhe and insertion of the XmaI/NheI digested PCR-product into the XmaI/NheI digested pCAGGS vector [28]. The resultant plasmid was digested with MlsI/Ecl136II and religated to yield the expression plasmid pCAGGS-HeV M. On the amino acid level the cloned sequence was 100% identical to HeV M accession number AEB21196. pCAGGS N-Strep HeV M was cloned by PCR amplification of the M ORF from pCAGGS-HeV M with the primer pair HeV MNhe/EclStrepHDM and insertion of the Ecl136II/NheI digested PCR product into Ecl136II/NheI digested pCAGGS. To generate pCAGGS C-Strep HeV M, the HeV-M coding sequence was PCR amplified from pCAGGS-HeV M with the primer pair HeV Mup/HeV MStrep and a BsrGI/NheI DNA-fragment was inserted into the BsrGI/NheI digested pCAGGS-HeV M. For cloning of pCAGGS-NiV M, a synthetic NiV M coding cDNA (Eurofins, Germany) coding for the NiV M protein (accession number NP_112025) was EcoRI/NheI digested and inserted into the pCAGGS.

The cds for human ANP32B was RT-PCR amplified as a 0.78 kb DNA-fragment from HEK293T RNA with primers ANP32Bfwsma/ANP32Btaaeco. The SmaI/EcoRI digested PCR product was cloned in the Eco47III/EcoRI digested pCtlGFP to generate pCtlANP32B. pCtlGFP is a mCherry-GFP fusion protein encoding derivative of pcDNA 3.1 (Invitrogen). The fluorescence protein coding sequences are separated by a (GGGGS)_3_ linker sequence (unpublished). The amino acid sequence of the cloned ANP32B was 100% identical to ANP32B accession number NP_006392. ANP32B was cloned by PCR amplification of the ANP32B cds from pCtlGFP with primers ANP32BEco/ANP32BNhe and insertion of the EcoRI/NheI digested PCR product into EcoRI/NheI digested pCAGGS. Accordingly, pCAGGS-HisANP32B was generated by PCR-amplification of the ANP32B cds with primers ANP32BNhe/RGShisANP from pCAGGS-ANP32B and insertion of an EcoRI/NheI DNA fragment into EcoRI/NheI digested pCAGGS. Detailed sequence information about primers and plasmids are available from the authors on request. Plasmid for expression of N-terminally tagged Strep-ANP (pCAGGS-StrepANP) was a gift from Linda Brunotte and Martin Schwemmle, Freiburg.

### DNA Transfection

DNA transfections into HEK293T cells were performed with polyethylenimine (PEI; Sigma-Aldrich). Briefly, for the transfection of 1×10^6^ cells in 3.5 cm dishes, 6 µg plasmid DNA were mixed with 9 µg PEI in 800 µl DMEM. After 20 min incubation at room temperature, the DNA-PEI mix was added to the cell cultures. After 3.5 h of incubation the medium was replaced by fresh medium. For plasmid transfections into A549 cells, 1 µg plasmid DNA was mixed with 3 µl FuGENE HD transfection reagent (Promega) in 20 µl OptiMEM I (Lifetechnologies) and was added to A549 cells seeded on coverslips.

### Protein Purification

For the purification of Strep-tagged M proteins 2×10^7^ HEK293T cells were transfected with 120 µg expression plasmid. 24 h later, cells were washed with PBS (w/o Ca^2+^ and Mg^2+^) and detached from the culture bottom by incubation with 5 mM EDTA in PBS (w/o Ca^2+^ and Mg^2+^). After centrifugation (5 min, 450 rcf, 4°C), the cell pellet was resuspended in 3 ml lysisbuffer (50 mM Tris-HCl pH 7.4, 150 mM NaCl, 2 mM CaCl_2_, 1% CHAPS or 0.5% Nonidet P-40 and 1x protease inhibitor cocktail (Roche)). After 1 h incubation at 4°C cell extracts were 5-times squeezed through syringe needles (d = 0.4 mm) and centrifuged (10 min, 450 rcf, 4°C). The supernatant was incubated over night with 500 µl StrepTactin Sepharose (50% (w/v) in lysisbuffer; IBA) at 4°C. Four washing steps with 400 µl wash buffer (50 mM Tris-HCl pH 7.4, 150 mM NaCl, 2 mM CaCl_2_) were performed prior to elution with 900 µl 1x elution buffer (100 mM Tris-HCl pH 8.0, 150 mM NaCl, 1 mM EDTA, 2.5 mM D-Desthiobiotin). Three elution fractions of 300 µl each were collected.

Purification of Strep-tagged ANP was performed 48 hrs after transfection of 6×10^6^ HEK293T according to the protocol described above.

### Protein Digestion

Proteins were digested in solution with porcine sequencing grade modified trypsin (catalogue number V5111, Promega) in 5 mM Tris-HCl pH 8.0 supplemented with 1 mM CaCl_2,_ for 16 h at 37°C using a substrate: enzyme ratio of 50∶1.

### Protein Identification

Digested samples were analysed on a platform consisting of a nLC system (EASY-nLC II, Bruker) connected to a Proteineer fcII sample spotting robot (Bruker), and an UltrafleXtreme MALDI-TOF/TOF instrument (Bruker). Peptide samples equivalent to 1–5 µg protein were diluted in 0.5% TFA and injected onto a NS-MP-10 loading/desalting column (C18-modified silica gel, 5 µm bead size, inner diameter 100 µm, length 20 mm; BioSphere), washed with 40 µl 0.05% TFA (5 µL/min) and eluted onto the analytical column (EASY-Column, 3 µm bead size, inner diameter 75 µm, length 10 cm, catalogue number SC200; Proxeon) at a flow rate of 300 nl/min, which was maintained throughout the following chromatographic run. A linear gradient of buffers A (0.05% TFA) and B (90% ACN, 0.05% TFA) was applied rising from 2% to 45% buffer B in 64 min and then to 100% B in 10 min. Every 10 s a fraction of 420 nl was collected on a 384-sample Anchor Chip target (Bruker) and mixed with saturated α-cyano-4-hydroxycinnamic acid (HCCA) in 90% ACN/0.1% TFA at a flow rate of 150 µl/h.

A maximum of 40 fragment spectra per spot was acquired from peaks with signal-to-noise ratios exceeding 5. Fragment spectra were passed to a Mascot server (version 2.3.02; Matrix Science) using the sequences of a *Homo sapiens* reference proteome set [29] as database for protein identification. The results of the database query were exported to ProteinScape software (Bruker) and protein identities reconstructed by the Protein Extractor feature in ProteinScape. Peptide and fragment mass tolerances were set to 25 parts per million (MS) and 0.7 Da (MS/MS). The minimum Mascot peptide score required to use MS/MS spectra for the compilation of proteins was 15. The significance level for the Mascot software was 0.98. Only proteins identified with at least one peptide exceeding the Mascot identity threshold score were reported. Optional methionine oxidation was allowed and the number of optionally missed cleavage sites was set to 2.

### Antibodies and Sera

For the generation of a polyclonal HeV M serum, recombinant histidin-tagged HeV M protein was expressed in SF9 insect cells and purified proteins were used for rabbit immunization. The resultant αHDM serum recognizes both, the M proteins of HeV M and NiV M. Polyclonal rabbit serum P160-5 against rabies virus phosphoprotein P has been described [30]. Antibodies ANP32B (G-12) and ANP32A/B Antibody (H-163) have been purchased from Santa Cruz Biotechnology, Inc. Monoclonal mouse antibody F45G5 against NiV M has been described before [31].

### Leptomycin B Treatment

For selective inhibition of Crm1-dependent nuclear export leptomycin B (LMB; Sigma-Aldrich) was added to cell cultures in a working dilution of 40 ng/ml cell culture medium. After 2 h of LMB treatment cells were fixed and conducted to indirect immunofluorescence analysis.

### Indirect Immunofluorescence Microscopy

For indirect immunofluorescence, monolayer cultures were fixed with 3% paraformaldehyde in PBS and permeabilized with 0.5% Triton-X100 in PBS. Immunodetection of HeV M was performed with polyclonal rabbit anti-HeV M serum (αHDM; 1∶200 in PBS). M protein in NiV infected cells was detected by combined use of anti-HeV M and the monoclonal mouse antibody F45G5 (dilution 1∶500 in PBS). ANP32B protein was detected with ANP32B (G-12) or ANP32A/B antibodies at dilutions of 1∶1000 in PBS. In case of ANP32B (G-12), 0.1% SDS was added during cell permeabilization. AlexaFluor 488 and Alexa Fluor 568 conjugated secondary antibodies were used (1∶1000 in PBS; Molecular Probes). All images were acquired with a Leica SP5 confocal laser scanning microscope without saturated pixels. Images were processed with the ImageJ software version 1.48b [32].

### Virus Infections

All experiments with live NiV were performed under biosafety level 4 (BSL-4) conditions at the Institute of Virology, Philipps University Marburg. The NiV Malaysia strain isolated from human brain propagated as described in [33] [34]. For NiV infection, confluent A549 cell monolayers that have been transfected for 24 h with mCherry-ANP32B were infected with NiV at a multiplicity of infection (MOI) of 0.5. After incubation for 1 h at 37°C, input virus was removed and cells were cultured in medium containing 2% FCS. At 20 h post infection, cells were inactivated and fixed in 4% paraformaldehyde for 48 h. Then, cells were permeabilized with methanol/acetone (1∶1) and immunostained using M-specific primary antibodies and AlexaFluor 488-labelled secondary antibodies. Nuclei were counterstained with DAPI. Samples were mounted in Mowiol and analyzed with a laser scanning microscope (Leica SP5). For Nipah virus growth curves, ANP-knockdown and control cells were infected with NiV at an MOI of 0.01. After 1 h at 37°C, input virus was removed, cells were washed three times and incubated in DMEM 2% FCS at 37°C. To analyze virus growth, samples from the supernatant were taken at 20, 24 and 45 h p.i., and titers were determined by the 50% tissue culture infectious dose (TCID_50_) method on Vero76 cells.

## Results

### Expression of Tagged HeV M

**Figure 1:**
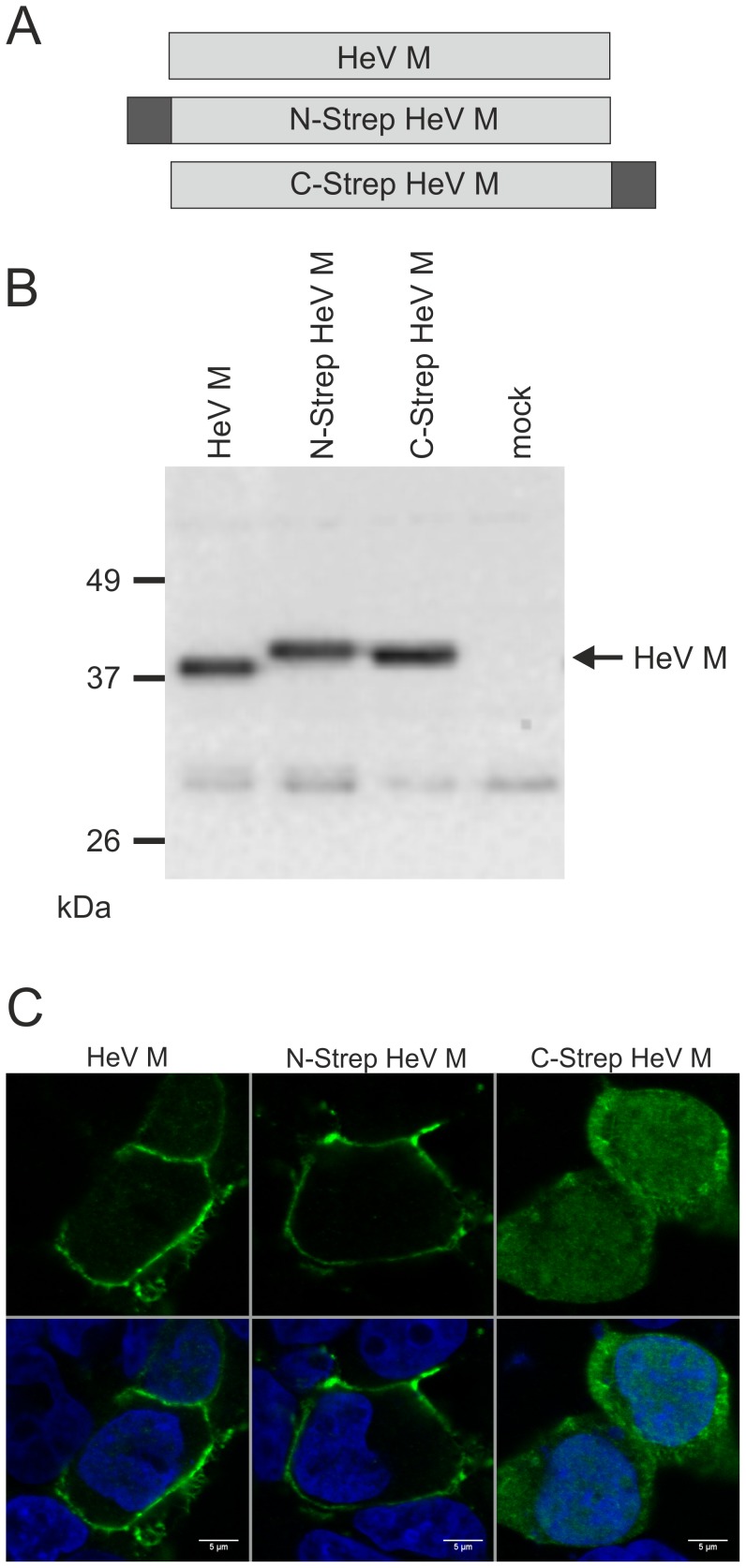
Strep-tagged HeV M proteins and expression in plasmid transfected HEK293T cells. (A) Schematic drawing of non-tagged HeV M and N- and C-terminally tagged HeV M proteins. The Strep-tag sequence is indicated by dark boxes. (B) Western Blot with HeV M specific serum confirming HeV M expression in plasmid transfected cells. (C) 16 h after transfection the cells were fixed and conducted to indirect immunofluorescence with HeV M specific serum and confocal laser scan analysis. No immunostaining was detectable in empty vector transfected cells (not shown). Nuclei were stained with Hoechst 33342 (blue). Scale bar: 5 µm.

In order to express HeV M for affinity purification of M containing protein complexes, plasmids coding for N- and C-terminally tagged HeV M proteins were generated (Fig. 1A). In both constructs, the 8 amino acid Strep-tag II sequence and a dinucleotide linker sequence were genetically fused to HeV M. Western Blot analyses with HeV M specific serum confirmed protein expression in plasmid transfected HEK293T cells. At 20 h post transfection, the protein levels of both N-Strep HeV M and C-Strep HeV M were comparable to non-tagged HeV M protein (Fig. 1B). The tags led to slightly increased molecular masses of N-Strep HeV M and C-Strep HeV M. Tagged HeV M was also detected with Strep-tag specific antibodies (not shown).

### Intracellular HeV M Distribution

The intracellular distribution of tagged and non-tagged HeV M in HEK293T was compared by confocal laser scanning microscopy. Cells were transfected with expression plasmids for HeV M, N-Strep HeV M and C-Strep HeV M and fixed after 16 h. Immunodetection with HeV M specific serum revealed that HeV M accumulated at the plasma membrane and only faint HeV M signals were detectable in the nucleus (Fig. 1C). Whereas N-Strep HeV M distribution was similar to that of untagged HeV M, C-Strep HeV M accumulation at the plasma membrane was not detectable, indicating that the C-terminal fusion interfered with membrane accumulation of C-Strep HeV M. Instead, stronger fluorescence was observed in the cytosol and the nucleus.

### Identification of ANP32B as a Potential Cellular Interactor of HeV M

**Figure 2:**
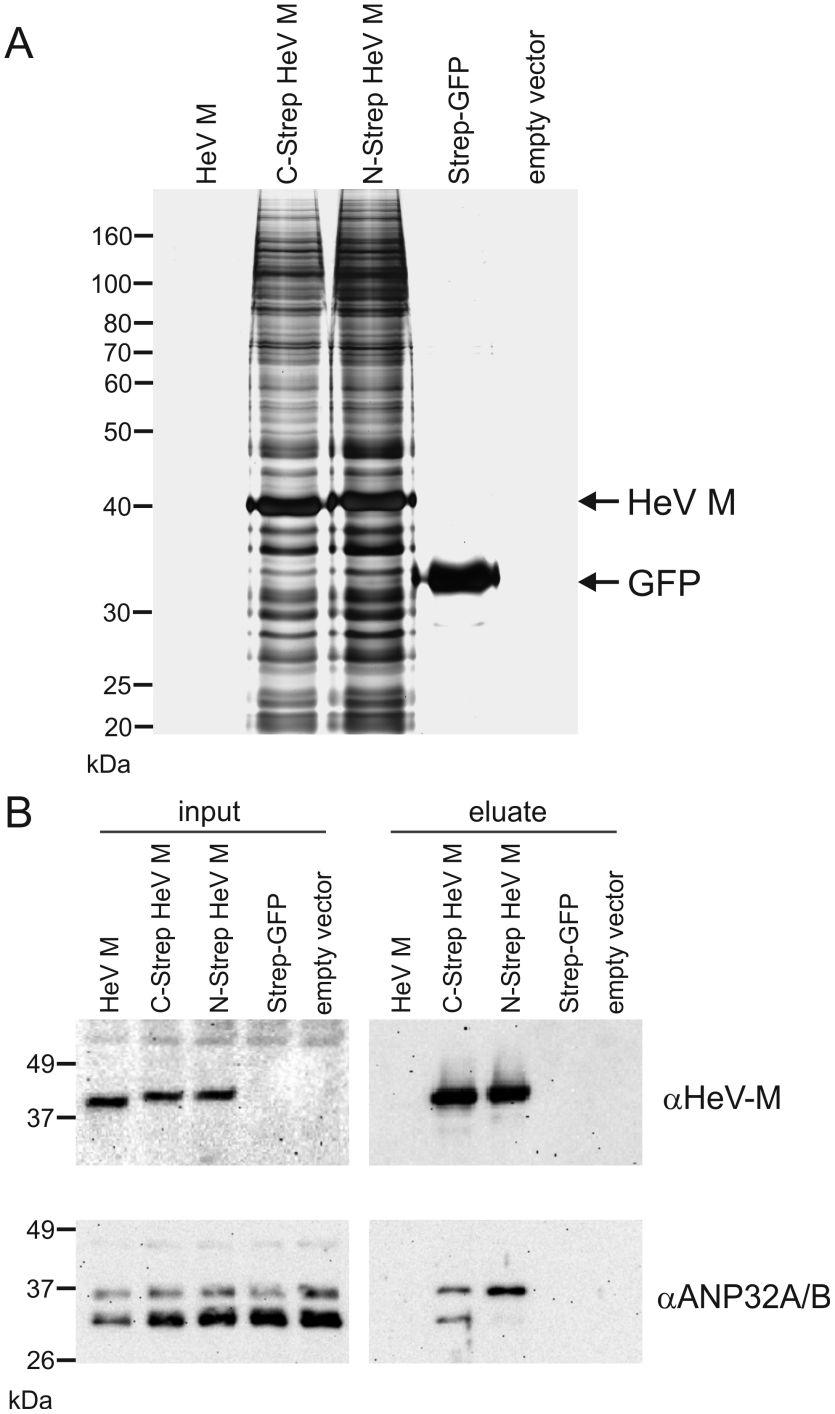
Co-purification of HeV M and ANP32B. Affinity-purification of tagged HeV M proteins from HEK293T cells was performed 24 h post transfection. Negative controls included untagged HeV-M, Strep-tagged GFP and empty vector transfected cells (empty vector). (A) Silver gel staining of Strep-tag purified protein samples. Only with tagged HeV M proteins multiple signals were detected with abundant signals at the expected molecular weights of HeV M. (B) Western Blot detection of HeV M and ANP32A/B in lysates from transfected cells (input) and purified protein samples (eluate). Both HeV M and ANP32 A/B were detected in samples from purified C-Strep HeV M and N-Strep HeV M, whereas no ANP32A/B was detected in purified samples from negative controls. Note: αANP32A/B serum recognizes both, ANP32B and ANP32A.

To identify cellular interactors of HeV M, the Strep-tagged HeV M proteins were purified 24 h after transfection of 2×10^7^ cells with the respective expression plasmids. Untagged HeV M and Strep-tagged GFP (Strep-GFP) were expressed as negative controls. Gel electrophoretic analysis of samples purified from N-Strep HeV M and C-Strep HeV M expressing cells revealed a dominant protein band at the expected molecular weight of 41 kDa and a number of co-purified proteins over a wide molecular weight range (Fig. 2A). Extraction of Strep-GFP resulted in a single band of lower molecular weight. Silver stainings further revealed blank gels from cells expressing non-tagged HeV M or transfected with an empty vector. The identity of the purified HeV M proteins was confirmed by western blotting (Fig. 2B) and mass spectrometry (not shown). The specificity of the affinity extraction was confirmed by the indicated negative controls.

Cellular proteins present in N-Strep HeV M and C-Strep HeV M complexes were also identified by mass spectrometry. After subtraction of proteins that were also identified in the control samples (Strep-GFP or empty vector) and application of stringent parameters for protein identification, GO-annotation analyses revealed that the majority of binding proteins represented ribosomal or ribosome-associated proteins (not shown), indicating the presence of M in ribosomal protein complexes.

Additionally, ANP32B (Acidic leucine-rich nuclear phosphoprotein 32 family member B, also designated as PHAPI2 or SSP29) protein was identified in C-Strep HeV M samples with high confidence in all three independent experiments, whereas N-Strep HeV M only led to identification of ANP32B in one out of three experiments. Four of the 10 peptides assigned to ANP32B were proteotypic for this protein so that ANP32B was unambiguously identified as part of the complex. Of the remaining six peptides, four were also present in ANP32A. The fragment spectra of the remaining two peptides could as well be assigned to two isobaric tryptic peptides of ANP32A with leucine/isoleucine exchanges in comparison to the sequence of ANP32B. Thus, the presence of ANP32B in the complex formed by HeV M was clearly shown but a concomitant enrichment of ANP32A could not be proved nor excluded by mass spectrometry. No ANP32-specific peptides were identified in the control samples.

The identity of the purified HeV M proteins was confirmed by western blotting (Fig. 2B). Whereas matrix protein was detectable in cell lysates from HeV M, C-Strep HeV M and N-Strep HeV M expressing cells, after affinity purification only the tagged M proteins remained detectable. Notably, with a serum that recognized both, the A and B members of the ANP32 family (αANP32A/B), two molecular weight forms at approximately equal levels were detected in purified C-Strep HeV M samples, whereas in N-Strep HeV M samples the upper form was abundant. Western blots with ANP32B specific serum identified the lower molecular weight signal as ANP32B (not shown). Decreased levels of the lower signal in N-Strep HeV M samples indicated interference of the N-terminal tag with binding to ANP32B.

### ANP32B-dependent HeV M Precipitation

**Figure 3:**
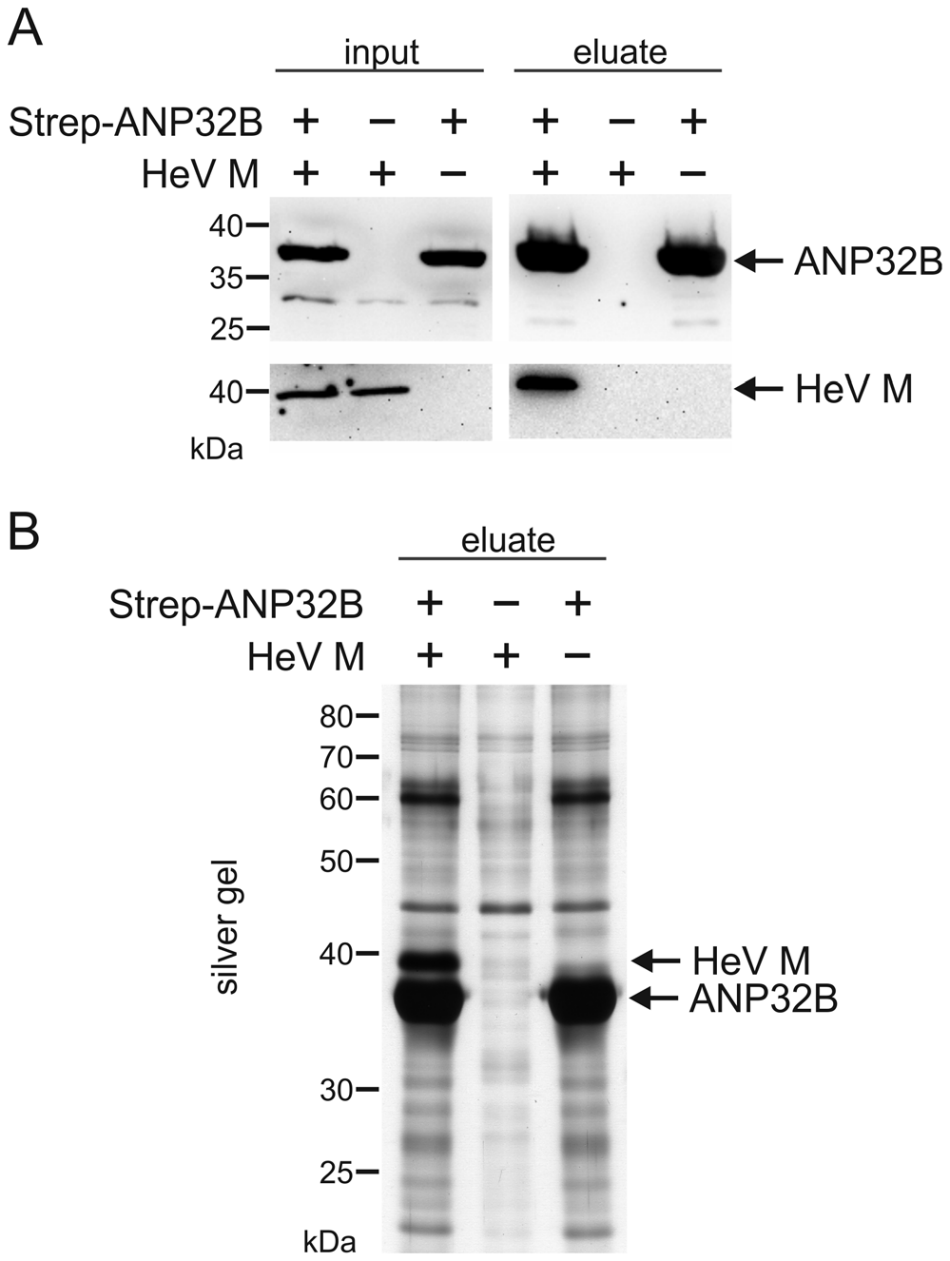
ANP32B-dependent precipitation of HeV M. (A) Western Blot detection of ANP32B and HeV M in cell lysates (input) and after purification of Strep-ANP32B (eluate). (B) silver gel analysis of purified protein samples. The identities of abundant signals at 40 and 35 kDa were confirmed by mass spectrometry as HeV M and ANP32B, respectively.

To further confirm a specific interaction of ANP32B with HeV M protein, co-purification of untagged HeV M protein with streptagged ANP32B was performed. Western blot analyses with samples from affinity purifications revealed specific co-purification of untagged HeV M with Strep-ANP32B (Fig. 3A) further supporting a direct or indirect physical interaction between HeV M and ANP32B. Notably, selective co-purification of untagged HeV M further confirmed, that the C-terminally added tag in C-Strep HeV M did not lead to artificial binding of ANP32B in Figure 2.

To assess whether HeV M protein was purified in stoichiometric amounts, silver gel analysis of purified protein samples was performed. Only in samples form cells expressing both, HeV M and Strep-ANP32B a strong signal at the expected molecular weight of 40 kDa for M was detected whereas an abundant signal at ∼35 kDa was present in both samples from ANP32B expressing cells (Fig. 3B). The identities of the detected proteins were confirmed by mass spectrometry. These data not only confirmed specific co-purification of untagged HeV M to ANP32B, but also reveal that the viral M protein can be co-purified with ANP32B at remarkable stoichiometric amounts.

### ANP32B Dependent Nuclear Accumulation of HeV M

**Figure 4:**
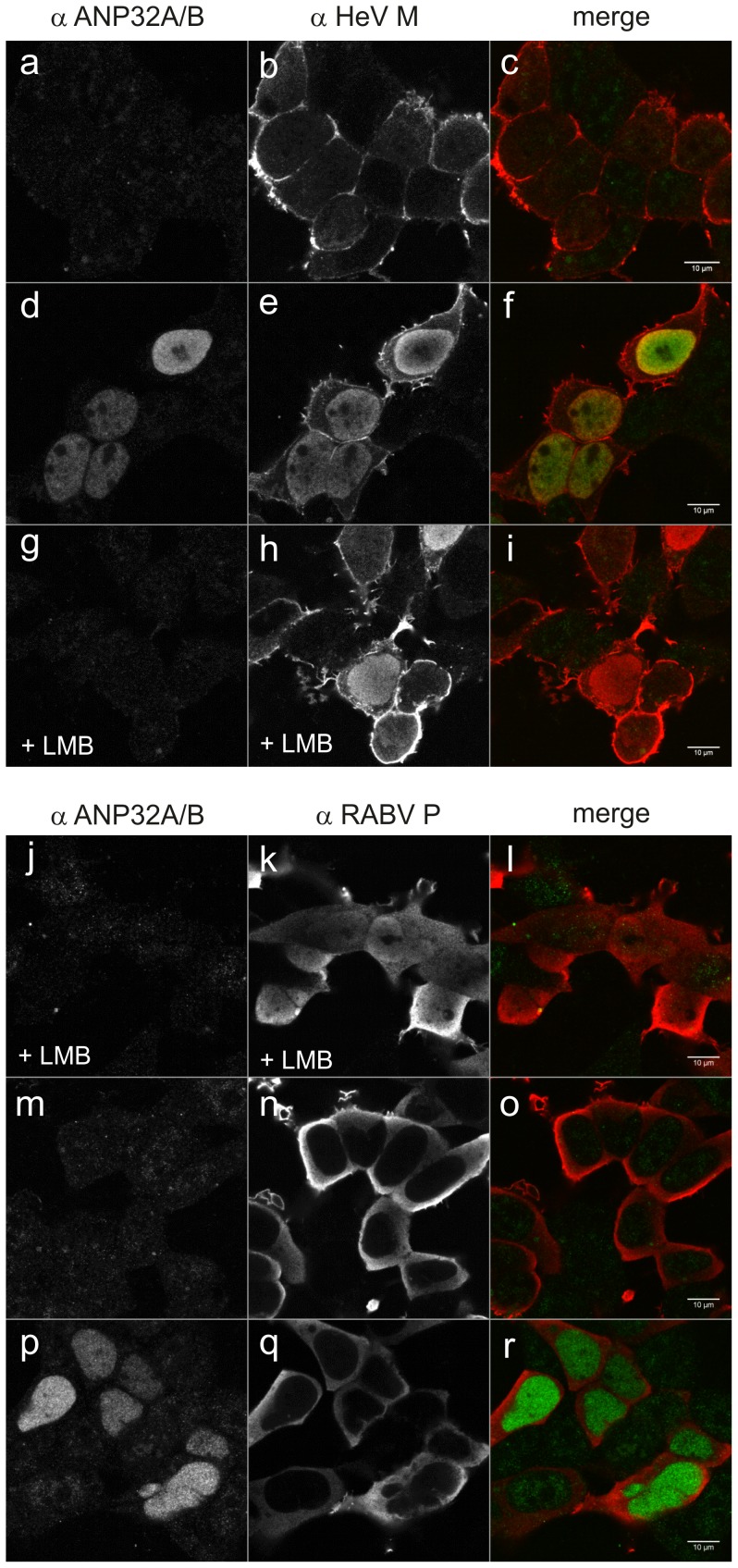
Over-expression of ANP32B results in specific nuclear accumulation of HeV M. (a–c) No nuclear accumulation was detectable in HEK 293T cells in the absence of over-expressed ANP32B. (d–f) At ANP32B over-expression, HeV M accumulated in the nucleus. (g–i) Leptomycin B led to nuclear accumulation of HeV M without over-expression of ANP32B. (j–l) Leptomycin B led to nuclear accumulation of rabies virus P. (m–o) In the absence of Leptomycin B, rabies virus P (RABV P) was not detected in the nucleus. (p–r) ANP32B over-expression did not induce nuclear accumulation of RABV P.

To assess whether ANP32B is functionally linked with HeV M, we analyzed whether ANP32B over-expression affects the intracellular trafficking of HeV M. For this purpose, untagged HeV M and ANP32B proteins were expressed in transfected HEK293T cells. After 24 h, cells were fixed and immuno-stained with ANP32A/B and HeV M specific sera (Fig. 4). In the absence of ANP32B expression plasmid, no nuclear M accumulation was detectable (Fig. 4 a–c). In contrast, co-expression of ANP32B and HeV M led to a strong accumulation of HeV M in the nucleus (Fig. 4 d–f), indicating that the intracellular localization of HeV M was altered in an ANP32B-dependent manner. It has been proposed that nuclear export of NiV M depends on chromosome region maintenance 1 (Crm1) [11]. In agreement with that, nuclear accumulation of HeV M was also induced by the Crm1-export inhibitor Leptomycin B (LMB) (Fig. 4 g–i), indicating that Crm-1 dependent export at least in part is also involved in nucleo-cytoplasmatic trafficking of HeV M. As ANP32B-dependent nuclear M retention could be a result of a more general effect of ANP32B on Crm1-dependent nuclear export, the specificity of the ANP32B-dependent nuclear retention of HeV M was checked with rabies virus P protein as a control. Rabies virus P is known to shuttle through the nucleus, using the Crm1 export pathway [35]. In the absence of LMB (Fig. 4 m–o) no nuclear P was detected. After LMB treatment P accumulated in the nucleus, confirming Crm1 dependent shuttling of P (Fig. 4 j–l). Most importantly, Crm1-dependent export of P was not inhibited by ANP32B (Fig. 4 p–r). These data showed that ANP32B over-expression does not lead to a general block in Crm1 dependent nuclear export. We therefore conclude that nuclear retention of HeV M is a specific effect of ANP32B on the HeV M nucleo-cytoplasmic trafficking process.

### ANP32B Affects the Intracellular Localization of both HeV M and NiV M

**Figure 5:**
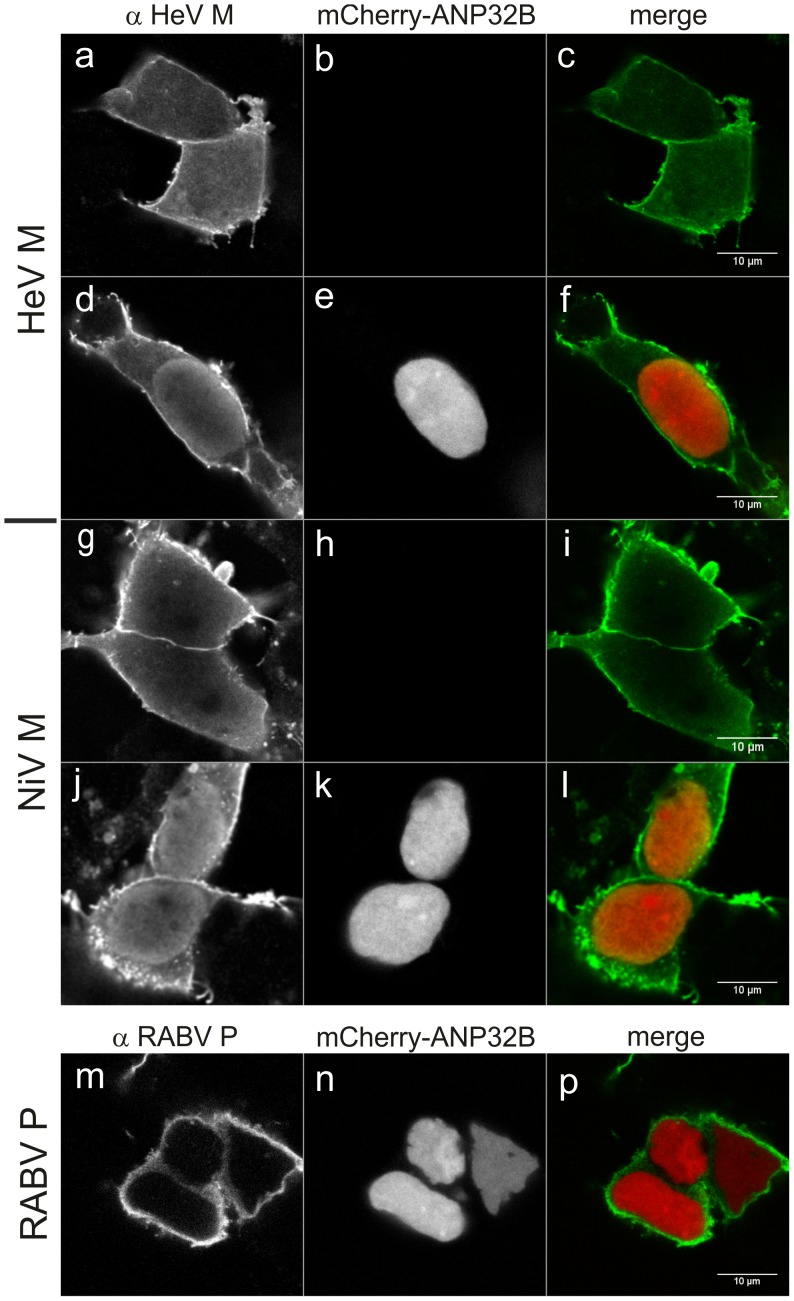
ANP32B dependent nuclear accumulation of NiV M protein. Co-expression of HeV M and NiV M with mCherry-tagged ANP32B in HEK293T cells resulted in nuclear accumulation of both virus proteins (d–f and j–l, respectively), whereas no nuclear accumulation of HeV M and NiV M was detectable in the absence of mCherry-ANP32B (a–c and g–i, respectively). No nuclear accumulation was detected for RABV P (m–p).

To elucidate whether NiV M is also affected by ANP32B, both henipavirus matrix proteins were co-expressed with fluorescence tagged ANP32B. In presence of mCherry-ANP32B both HeV and NiV M accumulated in the nucleus (Fig. 5d–f and j–l, respectively), whereas no nuclear accumulation was observed in the absence of mCherry-ANP32B (Fig. 5a–c and g–i). Nucleo-cytoplasmic trafficking of rabies virus P protein was not inhibited by mCherry-ANP32B. We conclude that HeV M and NiV M are similarly affected by ANP32B in their nucleo-cytoplasmic trafficking.

### ANP32B-dependent Nuclear Retention of M in Nipah Virus Infected Cells

**Figure 6:**
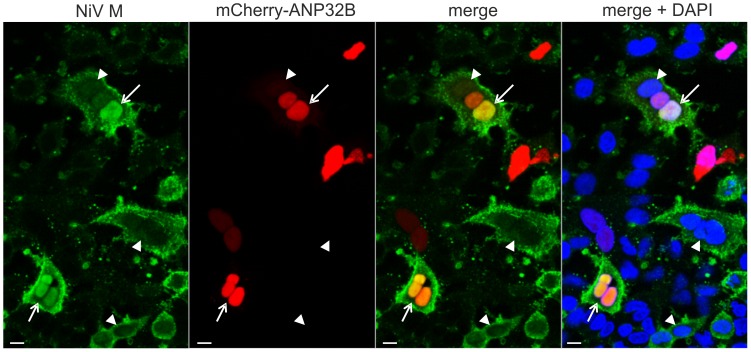
ANP32B-dependent nuclear retention of M in Nipah virus infected cells. After infection of mCherry-ANP32B expressing A549 cells at an MOI of 0.5, nuclear accumulation of NiV M was observed (arrows). In contrast, no accumulation was detectable in nuclei (DAPI-stained; blue) without mCherry-ANP32B (arrowheads). Shown are composites of two images. Scale bar: 10 µM.

As nuclear accumulation of the matrix proteins could be a result of isolated M expression outside the virus context, the effect of ANP32B on the intracellular distribution of NiV M was tested in Nipah virus infected cells. To this end, mCherry-ANP32B expressing A549 cells were infected with Nipah virus at an MOI of 0.5 at 24 h after transfection. After 20 h of infection, nuclear accumulation of NiV M was only detectable in mCherry-ANP32B expressing cells (Fig. 6, arrows), whereas no nuclear accumulation was observed in cells devoid of mCherry-ANP32B (Fig. 6, arrowheads). Remarkably, even in virus induced syncytia that harbored mCherry-ANP32B positive and negative nuclei, nuclear accumulation of M correlated with mCherry-ANP32B fluorescence, emphasizing the functional link between ANP32B and NiV M. Verification of the ANP32B-dependent henipavirus M protein retention in Nipah virus infected cells provide strong evidence that ANP32B indeed is a nuclear target of henipavirus matrix proteins that affects nucleo-cytoplasmic trafficking in the context of henipavirus infections.

### Virus Release is not Inhibited by shRNA Knock-down of ANP32B Expression

**Figure 7:**
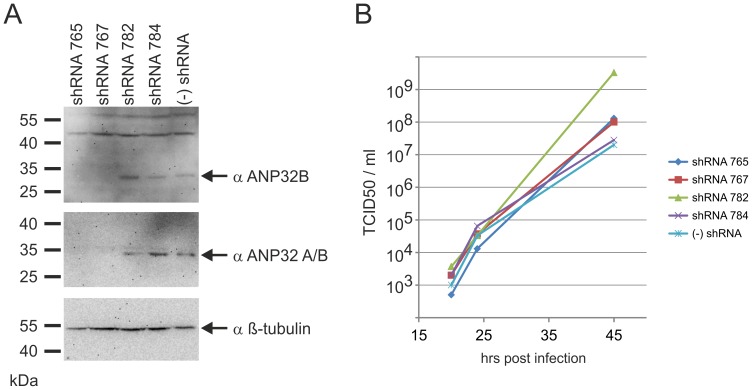
Virus release is not inhibited by shRNA knock-down of ANP32B expression. (A) Western Blots confirmed knock down of ANP32B protein in HEK-293T cells that stably express shRNAs against ANP32B mRNA (shRNA 765 and 767). Control cells shRNA 782 and 784 expressed irrelevant shRNAs and in authentic HEK293T cells ((−) shRNA) no shRNAs were expressed. (B) Growth curves for Nipah virus revealed that Nipah virus production was not affected by the shRNA knock down.

Since it has been published that nucleo-cytoplasmic shuttling of Nipah virus M protein is important for virus budding [11] we tested whether the release of Nipah Virus was affected by shRNA knock-down of endogenous ANP32B. We therefore generated two stable HEK293T cell lines (shRNA 765 and 767) in which endogenous ANP32B levels were down-regulated, as confirmed by western blots analysis with two different ANP32B specific sera (Fig. 7A). In contrast to the shRNA 765 and 767 cells, cells expressing irrelevant shRNAs (shRNA 782 and 784) exhibited ANP32B levels comparable to non-modified HEK 293T cells ((−) shRNA).

Nipah virus infection of the shRNA knock-down cells at an MOI of 0.01 and virus titration at 20, 24 and 45 hrs post infection revealed, that the ANP32B knock-down in shRNA 765 and 767 cells did not affect virus release (Fig. 7B). Thus we conclude that an interaction of M with ANP32B is not essentially involved in virus replication or budding and suspect that targeting of nuclear ANP32B by henipavirus M proteins more likely affects cellular ANP32B functions that might be involved in the regulation of cell responses to virus infections.

## Discussion

We identified cellular acidic leucine-rich nuclear phosphoprotein 32 family member B (ANP32B) as a nuclear target of henipavirus matrix proteins. Although the detailed role of a direct or indirect interaction with ANP32B in virus replication remains elusive, multiple functions of ANP32B in cellular mRNA export, gene expression regulation and apoptosis inhibition are known. For instance, ANP32B binds to Hu-antigen R (HuR)-mRNA complexes and, as an adaptor protein, recruits specific mRNAs to the Crm1 export machinery. Interestingly, stimulus driven export of CD83 mRNAs in dendritic cells [23] and also export of foamy virus are ANP32B-dependent [24]. In addition to the adaptor function of ANP32B in Crm1-dependent nuclear export, ANP32B is also known to regulate cellular promoters [26] and, as a direct caspase-3 substrate, inhibits caspase-3 dependent apoptosis induction [27]. The identification of the multifunctional ANP32B as a nuclear target of HeV supports novel hypotheses concerning the biological role of nuclear-cytoplasmic trafficking of henipavirus M proteins in the viral life cycle.

Mass spectrometry identification of ANP32B-specific peptides in purified protein samples from N-Strep HeV M and C-Strep HeV M expressing cells and subsequent western blot confirmation (Fig. 2B) supported the idea that ANP32B indeed is a cellular target of HeV M. Western Blot detection of two molecular masses with αANP32A/B serum (Fig. 2B) and identification of the lower signal as ANP32B led to the assumption that both ANP32B and the highly similar ANP32A were associated with HeV M. However, although the detection of two molecular weight forms correlated with a previous work demonstrating that ANP32B migrates faster than ANP32A in SDS-PAGE [36], by consideration of the ANP32-specific peptide masses neither the presence nor the absence of ANP32A in HeV M was confirmed. The different ANP32 western blot signal patterns thus either could rely on different affinities of the tagged HeV M proteins to the A and B members of the ANP32 family or on differential binding to variable ANP32B molecular weight forms. It is also conceivable that the introduced tags modified nuclear export kinetics of M protein. Consequently, increased nuclear C-Strep HeV M levels could have resulted in more efficient binding of ANP32B by C-Strep HeV M. However, highly efficient and specific co-purification of untagged HeV M with ANP32B (Fig. 3) in addition to ANP-32B dependent nuclear accumulation of untagged HeV and NiV M proteins (Fig. 4, 5 and 6) strongly supports that even untagged henipavirus M proteins interact with ANP32B.

While the dissection of possible interactions between HeV M and ribosomal complexes still needs to be performed in further studies, from co-purification experiments and ANP32B-dependent re-distribution of both henipavirus M proteins, our data suggest a functional link between henipavirus M proteins and ANP32B and conclude that ANP32B indeed is a nuclear target of henipavirus M proteins. Nuclear retention of both HeV and NiV M by ANP32B (Fig. 4 and 5) strongly supports the hypothesis that either nucleo-cytoplasmic trafficking of henipavirus M proteins is regulated by ANP32B or that M influences cellular functions of ANP32B. Nuclear retention of both matrix proteins also revealed that the interaction with ANP32B is conserved among these henipavirus M proteins, which exhibit 90% amino acid identity at the amino acid level. Further experiments with Cedar Virus (CeV), a less related member of the henipavirus genus, or other paramyxovirus matrix proteins may reveal whether targeting of ANP32B is conserved within the complete henipavirus genus and whether the identified virus-host interaction is more widespread within the paramyxovirus family.

Localization and interaction studies with plasmid-encoded viral M proteins cannot simply be transferred to the viral context. Here, other viral proteins are present that can interact with M thereby influencing its intracellular localization and/or its interaction with cellular components. Therefore, it was important to verify the effect of ANP32B on the intracellular distribution of M in the full context of a virus-infected cell. ANP32B-dependent nuclear retention of matrix protein in NiV infected cells (Fig. 6) indeed further suggested that the matrix protein and ANP32B are linked even in presence of the complete virus proteome and virus-induced cytopathic effects.

Nucleo-cytoplasmic trafficking of henipavirus M proteins appears enigmatic. Two leucine-rich sequence motifs have been identified to be involved in NiV M nuclear export. Moreover it has been shown that these motifs are not sufficient for nuclear export since mono-ubiquitinylation of NiV M at a defined lysine residue was required for nuclear export [11] indicating a conditional Crm1-dependent nuclear export of which key cellular factors have not yet been identified. Whereas Crm1-dependent nuclear export of NiV M has been concluded from mutagenesis studies by the identification of two leucine-rich nuclear export sequences [11], we demonstrated here that HeV M also requires Crm1-dependent nuclear export mechanisms during nucleo-cytoplasmic trafficking by direct inhibition of the nuclear export of HeV M with Crm1-specific inhibitor Leptomycin B (Fig. 4g–i). A less complete nuclear accumulation of HeV M in presence of Leptomycin B as compared to RABV P (Fig. 4d–f) could be the result of different kinetics of HeV M and RABV P trafficking or due to tethering of cytoplasmic M protein at cellular membranes.

To assess the specificity of ANP32B-dependent inhibition of the Crm1-dependent export of the henipavirus matrix proteins, it was important to show that ANP32B over-expression did not lead to a general inhibition of the Crm1 export machinery. Indeed, in ANP32B over-expressing cells, the adaptor function of ANP32B in mRNA export processes, which involves direct binding of Crm1 [23], could block protein export by competition for Crm1 [37], [38]. Introduction of rabies virus P as a control for ANP32B-independent nuclear export therefore was essential to demonstrate the specificity of the ANP32B-dependent nuclear retention of the henipavirus proteins (Fig. 4) and to draw the conclusion that ANP32B is a specific nuclear target of henipavirus matrix proteins.

Several virus M proteins recruit cellular ubiquitin-ligases by L (late) domains. Ubiquitin-ligases may be involved in mono-ubiquitinylation of M, and in M protein mediated virus budding (reviewed in [39]). In NiV M classical L-domain motifs are absent and L-domain functions of the known motifs YMYL and YPLGVG, although important for budding, remain unclear [9], [10]. As point mutations within the YPLGVG lead to nuclear accumulation of NiV M [10], it is conceivable that ubiquitin-ligases or other proteins involved in mono-ubiquitinylation bind via these motifs and induce nuclear export. With ANP32B we have identified a nuclear target of henipavirus M proteins that affects nucleo-cytoplasmic trafficking of NiV and HeV M. Although it is not known whether ANP32B affects the post-translational modification of the M proteins (e.g. mono-ubiquitinylation), ANP32 familiy members have been described as potential substrates for small ubiquitin-like modifier (SUMO) 2 [40], providing a possible link to the cellular sumoylation machinery and posttranslational modification of M during nucleo-cytoplasmic trafficking. Experimental data that could support involvement of sumoylation in M protein trafficking, however, are not available so far.

As ANP32B is directly involved in nuclear mRNA export processes [23], [41], it is also conceivable that ANP32B is directly involved in nuclear export of henipavirus M proteins. Although we show here that over-expression of both, untagged and tagged ANP32B results in nuclear accumulation of M, we cannot exclude the possibility that endogenous ANP32B at physiological levels supports efficient conditional export of M through Crm1. On the other hand, ANP32B may tether M in the nucleus by interactions whose biological roles could be independent of M export processes.

Interference of henipavirus M proteins with ANP32B functions in cellular mRNA export, gene expression regulation and apoptosis regulation may contribute to efficient virus replication. For example, by serving as an adaptor between RNA binding protein HuR and Crm1, ANP32B is involved in Crm1-dependent CD83 mRNA export processes after T-cell activation [23]. Although a direct involvement of ANP32B has not yet been shown, also mRNA export of other T-cell activation dependent genes is Crm1-dependent [42], leading to the assumption that the ANP32B adaptor could play a central role in the regulation of activated T-cells and maybe in immune responses to viral infections. As productive infection of monocytes, CD6+CD8+ T lymphocytes and NK cells by NiV may support crossing of the blood brain barrier and neuroinvasion [43], modulation of T-cell responses by direct interference with T-cell activation-dependent gene expression control may represent an important step in virus replication and pathogenesis. Further experiments are required to assess whether a direct or indirect interaction of henipavirus M proteins with ANP32B not only is able to retain M in the nucleus but also affects ANP32B cellular functions in mRNA export. Interference of henipavirus M proteins with ANP32B-dependent apoptosis [27] or gene regulation [26] represents conceivable strategies of host cell manipulation by henipaviruses M proteins.

In the present study we identified ANP32B as a nuclear target of HeV and NiV M proteins. ANP32B may serve as a binding factor, either involved in the regulation of nucleo-cytoplasmic trafficking of henipavirus M proteins or in the targeted interference of henipavirus M proteins with cellular reactions to virus infections.
